# Supplementary material for: 2.7 Å cryo-EM structure of ex vivo RML prion fibrils
Source: Nat Commun. 2022 Jul 13;13:4004. doi: 10.1038/s41467-022-30457-7 (PMC9279362; doi:10.1038/s41467-022-30457-7)
Supplement: Supplementary file 1 — Supplementary Information [file 41467_2022_30457_MOESM1_ESM.pdf]

Supplementary Information

2.7 Å cryo-EM structure of ex vivo RML prion fibrils

Szymon W. Manka, Wenjuan Zhang, Adam Wenborn, Jemma Betts, Susan Joiner, Helen R. Saibil, John Collinge, Jonathan D.F. Wadsworth

Supplementary Figures

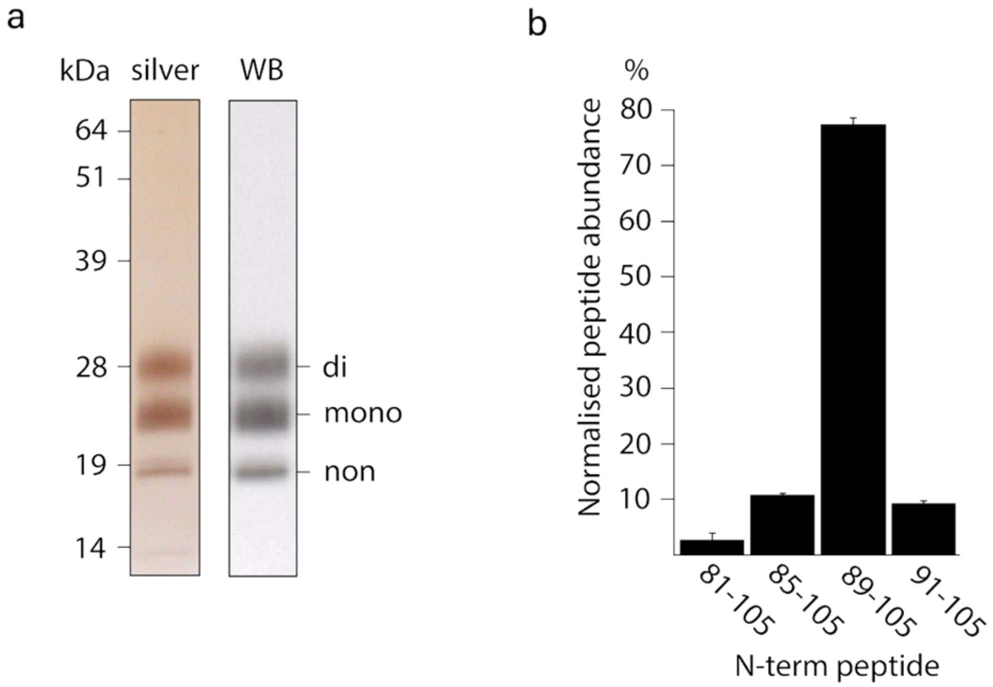

Supplementary Figure 1. **Biochemical characterisation of purified RML prion rods. a)** Silver-stained SDS-PAGE and western blot (WB) of purified RML rods; di, mono and non denote di-, mono- and non-glycosylated PrP bands, respectively. The samples were prepared as described in Methods. Uncropped and unprocessed scans are provided as a Source Data file. **b**) Determination of N-terminal PK-cleavage sites in purified RML prion rods by mass spectrometry. N-terminal PK-cleavage sites in PrP rods were determined by the targeted derivatization of α-amino groups with TMPP and subsequent analysis by mass spectrometry as described in Methods. Extracted ion chromatograms were generated for each TMPP-labelled peptide (mouse PrP residue ranges) and relative abundance was determined from their respective peak areas. Bars show mean ± standard deviation, three independent experiments. Associated raw data are provided as a Source Data file.

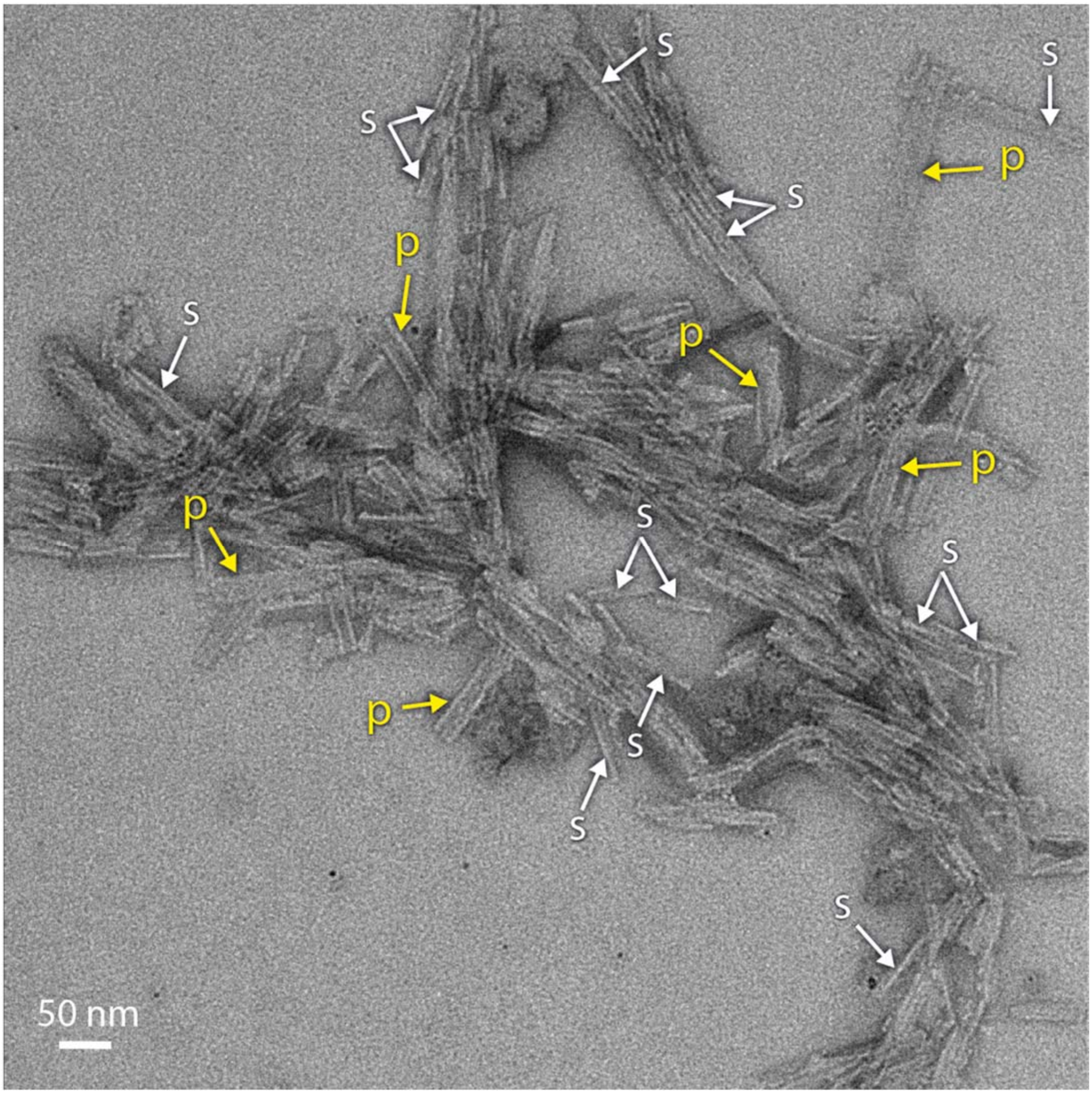

Supplementary Figure 2. Negative-stain EM of purified RML prion rods. Clear examples of single (s) and paired (p) fibrils are indicated. The aggregated (clumped) fibrils are difficult to interpret or quantify. The rods were stained with 2 % solution of NANO-W^{TM} stain (Nanoprobes) and imaged on a 120 kV Talos microscope (FEI/Thermo Fisher) with a 4k x 4k BM-Ceta camera.

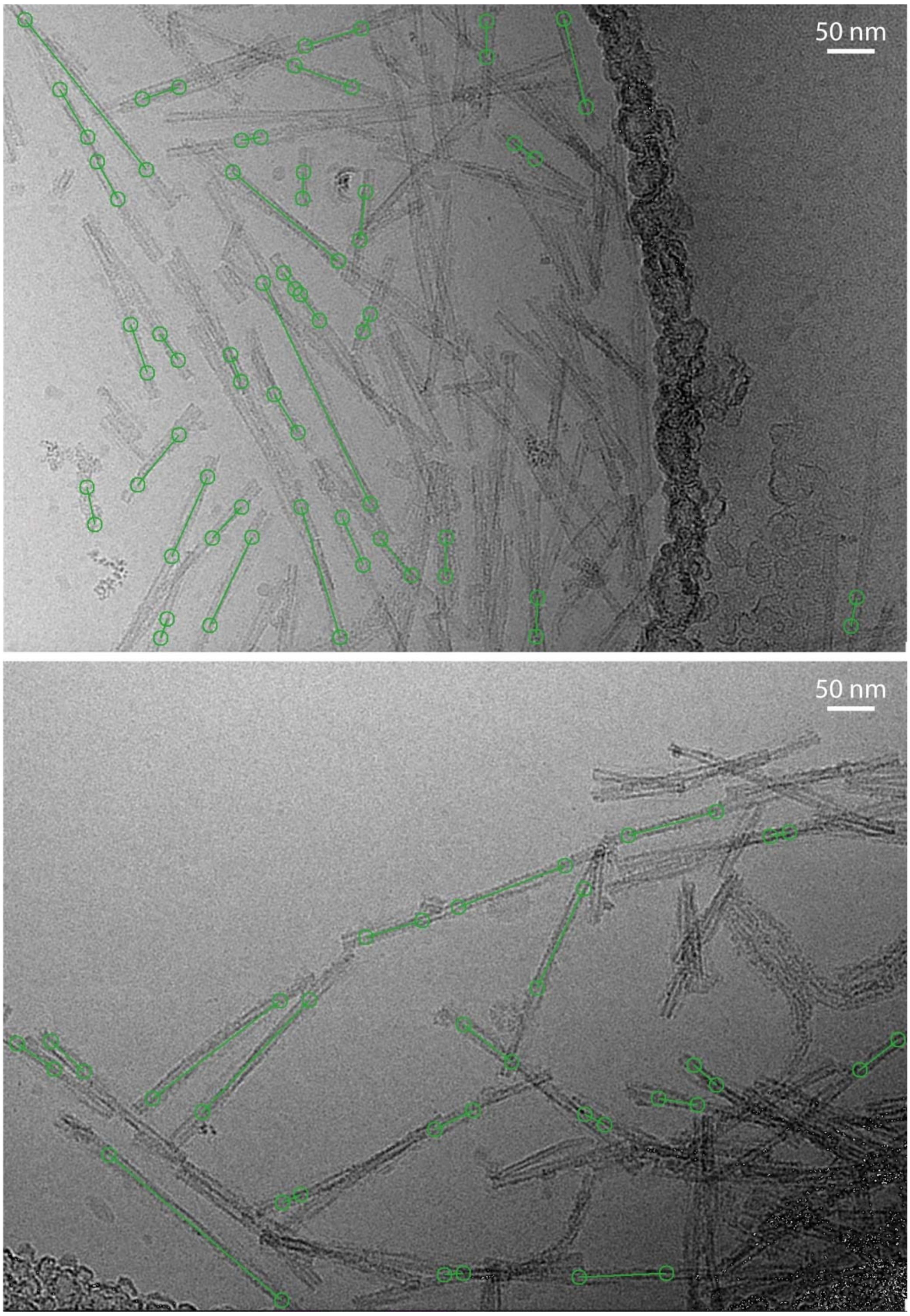

Supplementary Figure 3. Automated picking of RML rod particles with crYOLO. Shown are example raw cryo-micrographs (after movie frame alignment; collected on a Krios G3i microscope with a K3 camera (Gatan) as described in Methods) with example filament picking by crYOLO[1] (green circles for fibril start-end coordinates connected by a line). The programme mostly picked single fibrils and avoided clumped or bundled fibrils (as trained), but occasionally picked the unwanted narrower views of paired filaments or filaments on carbon support. These erroneously picked particles were excluded from further processing and 3D reconstruction through 2D and 3D classifications.

a

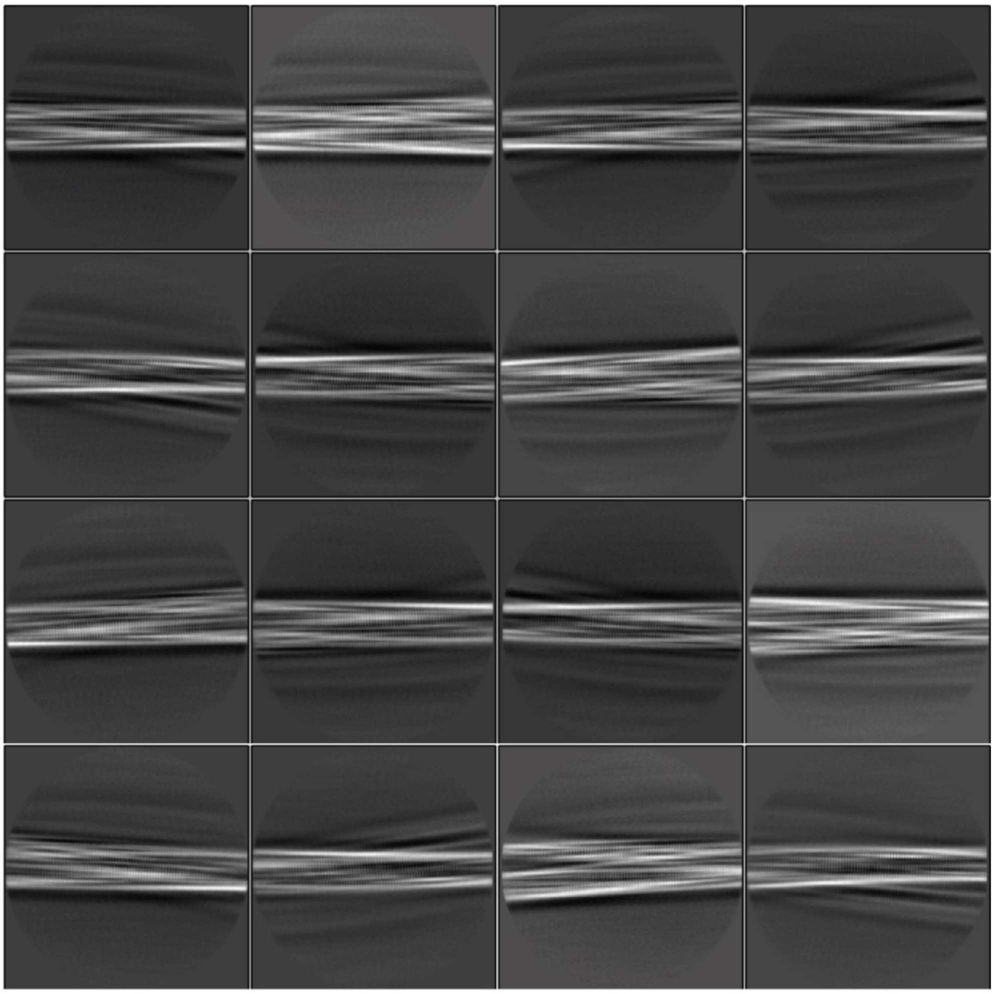

b

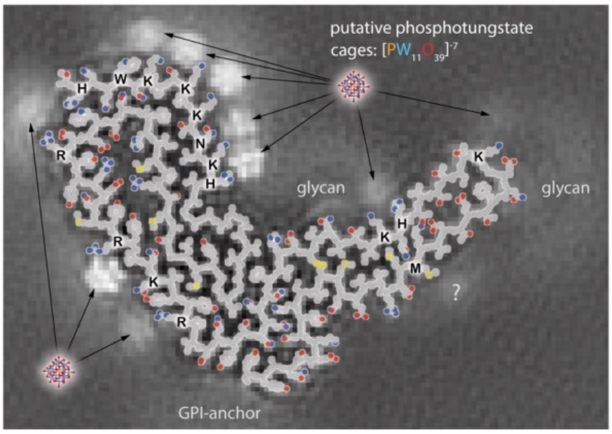

Supplementary Figure 4. 2D classification of RML prion fibrils and extended interpretation of their average cross-section. a) Gallery of representative 2D classes (box size: 409.7 x 409.7 Å). **b)** Single PrP subunit ball and stick model (backbone, grey; side chain atoms: C, grey; O, red; N, blue; S, yellow) superposed on the fibril 3D reconstruction cross-section image. Positively charged residues proximal to the putative phosphotungstate polyanions are labelled. Putative individual tungsten (W) atoms are resolved at most highly occupied binding sites (high densities). The bottom-right extra density is difficult to interpret (question mark) as it occupies an unusual position for phosphotungstate (near M side chain).

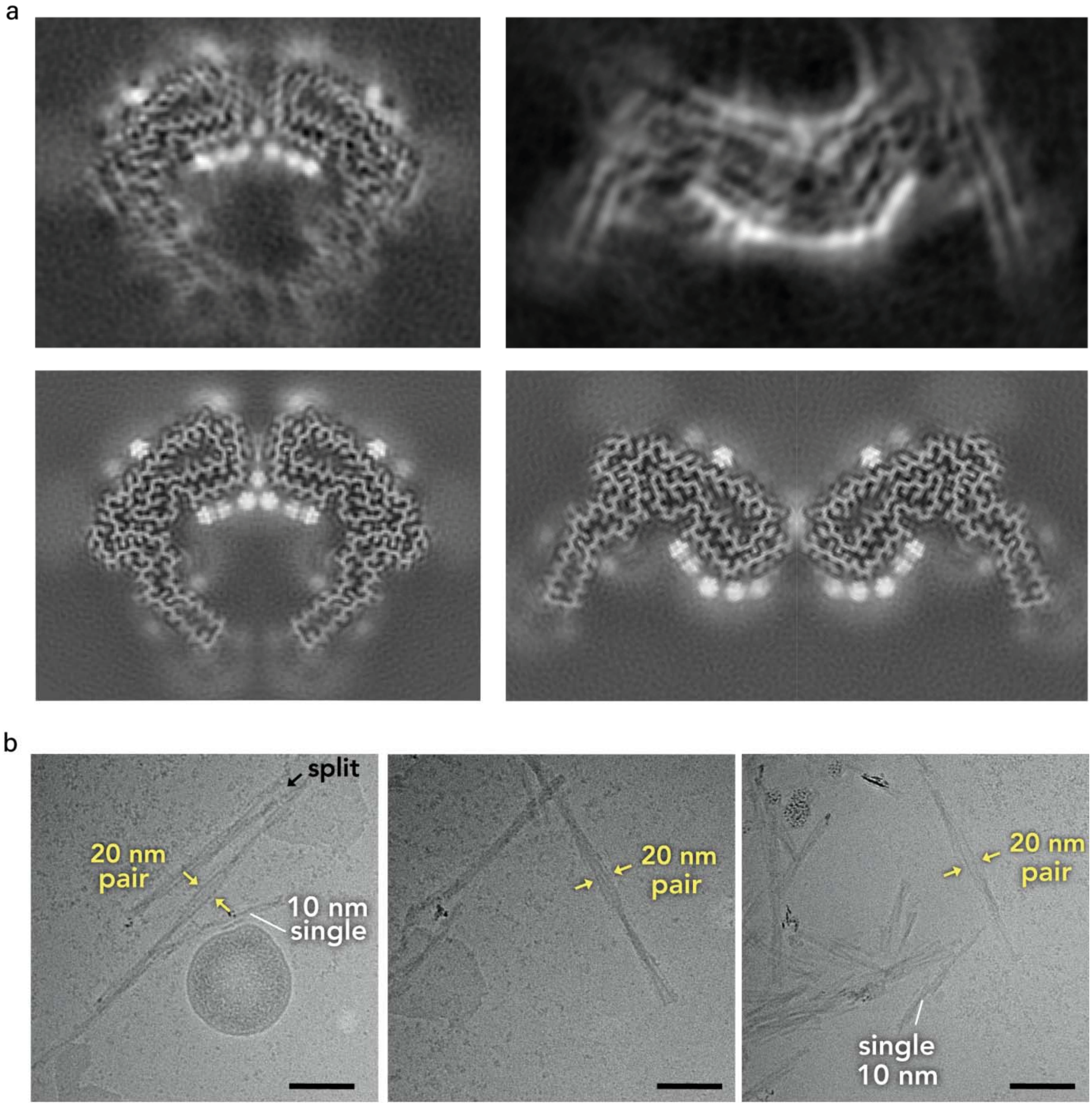

Supplementary Figure 5. Paired RML fibril assemblies. **a)** Top panels, central slices through low-resolution maps of the two types of paired RML protofilaments observed. Bottom panels, central slices through high-resolution maps of single RML protofilaments manually arranged according to the observed types of paired protofilament assemblies. **b)** Example cryo-EM images of RML rods purified without PTA. Both paired and single protofilament fibrils were observed in the absence of PTA. Micrographs were collected using a 120 kV Talos microscope with a tungsten filament and a BM-Ceta camera (Thermo Fisher). Scale bar, 100 nm.

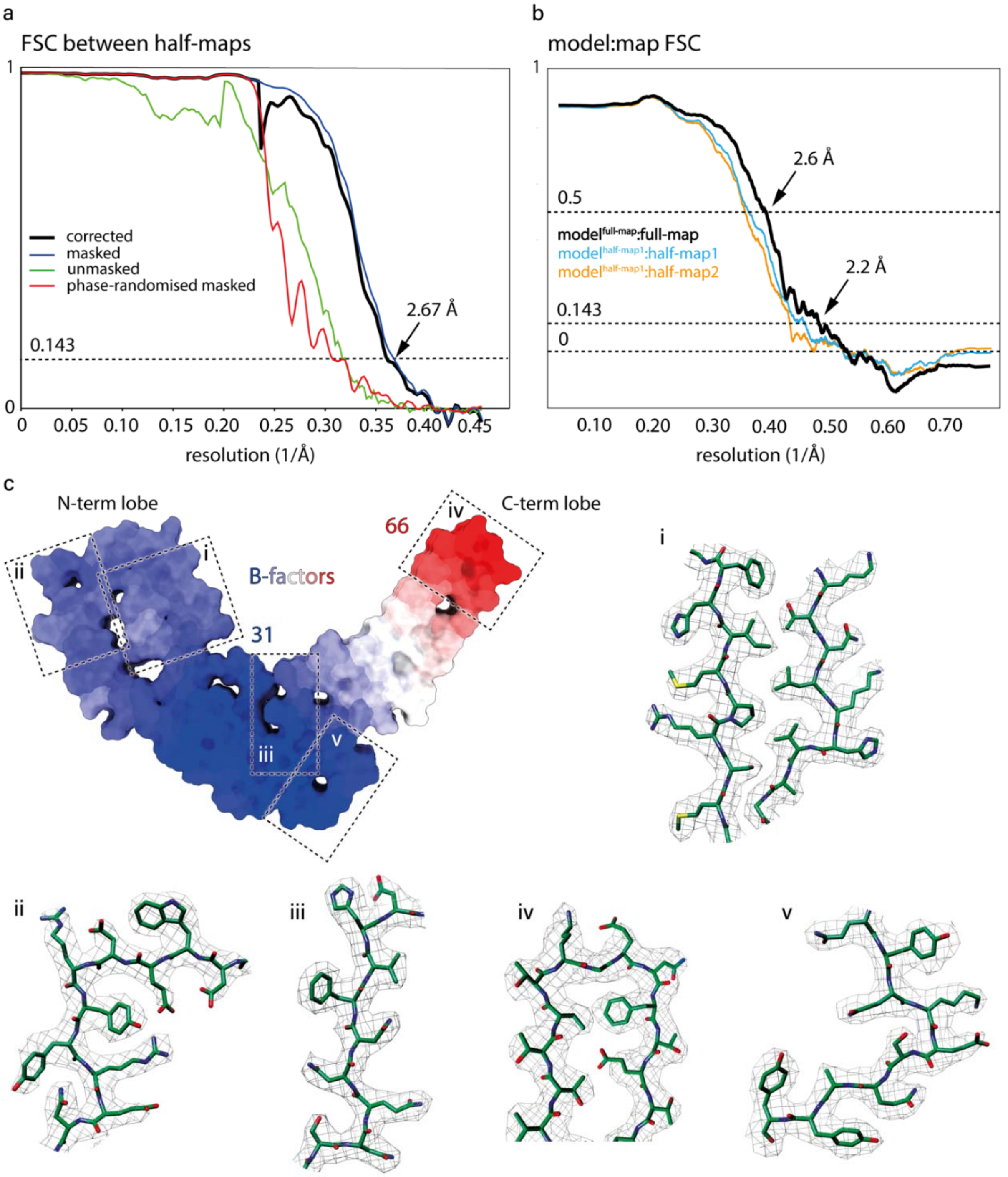

Supplementary Figure 6. Cryo-EM map resolution and model:map fit. a) Fourier Shell Correlation (FSC) plots for independent half-reconstructions, as outputted by Relion 3.1. The final plot (black line) is corrected for overfitting with high-resolution noise substitution. **b)** FSC plots for: the final model, refined using the full (combined) map, against that map (black line); a model shaken and refined using the first half-map against that map (blue); and the same model against the second half-map (orange). **c)** Atomic B-factor values colour-coded on the solvent-excluded model surface and close-up views (i-v) of model:map fit in selected regions (C, green; N, blue; O, red; S, yellow; map, wireframe).

Supplementary References

1. Wagner, T. et al. Two particle-picking procedures for filamentous proteins: SPHIRE-crYOLO filament mode and SPHIRE-STRIPER. *Acta Crystallogr D Struct Biol* **76**, 613-620 (2020).
