# Supplementary material for: 2.7 Å cryo-EM structure of ex vivo RML prion fibrils
Source: Nat Commun. 2022 Jul 13;13:4004. doi: 10.1038/s41467-022-30457-7 (PMC9279362; doi:10.1038/s41467-022-30457-7)
Supplement: Supplementary file 3 — Reporting Summary [file 41467_2022_30457_MOESM3_ESM.pdf]

Corresponding author(s): Helen Saibil, John Collinge and Jonathan Wadsworth

Last updated by author(s): Apr 4, 2022

Reporting Summary

Nature Portfolio wishes to improve the reproducibility of the work that we publish. This form provides structure for consistency and transparency in reporting. For further information on Nature Portfolio policies, see our Editorial Policies and the Editorial Policy Checklist.

Statistics

For all statistical analyses, confirm that the following items are present in the figure legend, table legend, main text, or Methods section.

n/a	Confirmed	
☐	☒	The exact sample size (*n*) for each experimental group/condition, given as a discrete number and unit of measurement
☐	☒	A statement on whether measurements were taken from distinct samples or whether the same sample was measured repeatedly
☒	☐	The statistical test(s) used AND whether they are one- or two-sided<br>*Only common tests should be described solely by name; describe more complex techniques in the Methods section.*
☒	☐	A description of all covariates tested
☒	☐	A description of any assumptions or corrections, such as tests of normality and adjustment for multiple comparisons
☒	☐	A full description of the statistical parameters including central tendency (e.g. means) or other basic estimates (e.g. regression coefficient) AND variation (e.g. standard deviation) or associated estimates of uncertainty (e.g. confidence intervals)
☒	☐	For null hypothesis testing, the test statistic (e.g. *F*, *t*, *r*) with confidence intervals, effect sizes, degrees of freedom and *P* value noted<br>*Give P values as exact values whenever suitable.*
☒	☐	For Bayesian analysis, information on the choice of priors and Markov chain Monte Carlo settings
☒	☐	For hierarchical and complex designs, identification of the appropriate level for tests and full reporting of outcomes
☒	☐	Estimates of effect sizes (e.g. Cohen's *d*, Pearson's *r*), indicating how they were calculated

Our web collection on statistics for biologists contains articles on many of the points above.

Software and code

Policy information about availability of computer code

Data collection	EPU 2 (Thermo Fisher)
Data analysis	Relion 3.1 (MRC-LMB, Cambridge), EMAN2 2.31 (Tang et al., 2007), crYOLO 1.6.0. (Wagner et al., 2019), Gctf 1.06 (Zhang, 2016), Coot 0.9.6 (Emsley et al., 2010), PHENIX 1.19.2 (Afonine et al., 2018), REFMAC5 5.5 (Murshudov et al., 2011), ISOLDE 1.2 (Croll, 2018), MolProbity 4.5.1 (Williams et al., 2018), UCSF Chimera 1.15 (Pettersen et al., 2004), UCSF ChimeraX 1.1.1 (Pettersen et al., 2021)

For manuscripts utilizing custom algorithms or software that are central to the research but not yet described in published literature, software must be made available to editors and reviewers. We strongly encourage code deposition in a community repository (e.g. GitHub). See the Nature Portfolio guidelines for submitting code & software for further information.

Data

Policy information about availability of data

All manuscripts must include a data availability statement. This statement should provide the following information, where applicable:

- Accession codes, unique identifiers, or web links for publicly available datasets
- A description of any restrictions on data availability
- For clinical datasets or third party data, please ensure that the statement adheres to our policy

The 3D cryo-EM density map was deposited into the Electron Microscopy Data Bank (https://www.ebi.ac.uk/pdbe/emdb) under accession no. EMD-13989 [https://www.ebi.ac.uk/pdbe/entry/emdb/EMD-13989] (Infectious mouse-adapted RML scrapie prion fibril purified from terminally-infected mouse brains). The corresponding atomic coordinates were deposited in the Protein Data Bank (https://www.rcsb.org) under PDB code 7QIG [https://doi.org/10.2210/pdb7QIG/pdb]. The entire cryo-EM dataset (all aligned micrographs) were deposited at EMPIAR, a public repository for electron microscopy data, under accession code EMPIAR-10992 [https://dx.doi.org/10.6019/EMPIAR-10992]. Infectious mammalian prion fibril (263K scrapie) was accessed from PDB under code 7LNA [https://

doi.org/10.2210/pdb7lna/pdb].

Field-specific reporting

Please select the one below that is the best fit for your research. If you are not sure, read the appropriate sections before making your selection.

- [x] Life sciences
- [ ] Behavioural & social sciences
- [ ] Ecological, evolutionary & environmental sciences

For a reference copy of the document with all sections, see nature.com/documents/nr-reporting-summary-flat.pdf

Life sciences study design

All studies must disclose on these points even when the disclosure is negative.

Sample size	Sample size (the number of particle images or segments) was not predetermined. We aimed to collect ~6,000 multi-frame movies and this was achieved. Satisfactory sample size is reflected in the final near-atomic and isotropic resolution of the 3D reconstruction, sufficient for building and refining an atomic model.
Data exclusions	In single-particle cryo-EM data processing so called 'bad particles' are excluded due to their obvious poor quality, which precludes their alignment with the consensus data. The sparse regions of micrographs where sample image quality is poor (for example, due to grid surface contamination giving rise to local noise in particle image or due to sample heterogeneity) would ideally be not selected for processing, but this cannot be avoided, especially when using automated particle picking, as in this study. Image processing algorithms reveal such poor particles as not classifiable under objective computational criteria into any biologically relevant class, which objectifies exclusion.
Replication	~200 infected mouse brains were combined to produce one brain homogenate. The final cryo-EM dataset combined images from 5 independent rounds of purification of RML fibrils from this brain homogenate. The purification method was reported in 2015 (Wenborn et al Sci Rep 2015). The method is robust and we have had no replication failures after >100 repetitions. The method has been independently used and replicated in other laboratories.
Randomization	Not relevant to the study, since only one homogeneous state was targeted.
Blinding	Classifications of particle images were performed computationally and therefore objectively. Blinding was not relevant for prion purification and sample characterization as there was only one type of prion sample.

Reporting for specific materials, systems and methods

We require information from authors about some types of materials, experimental systems and methods used in many studies. Here, indicate whether each material, system or method listed is relevant to your study. If you are not sure if a list item applies to your research, read the appropriate section before selecting a response.

Materials & experimental systems

n/a	Involved in the study	
[ ]	[x]	Antibodies
[ ]	[x]	Eukaryotic cell lines
[x]	[ ]	Palaeontology and archaeology
[ ]	[x]	Animals and other organisms
[x]	[ ]	Human research participants
[x]	[ ]	Clinical data
[x]	[ ]	Dual use research of concern

Methods

n/a	Involved in the study	
[x]	[ ]	ChIP-seq
[x]	[ ]	Flow cytometry
[x]	[ ]	MRI-based neuroimaging

Antibodies

Antibodies used	ICSM 35 mouse anti-PrP monoclonal antibody was used for western blotting in conjunction with alkaline-phosphatase-conjugated goat anti-mouse IgG secondary antibody (Sigma-Aldrich, Cat No A2179). ICSM 35 and ICSM 18 mouse anti-PrP monoclonal antibodies were used for determination of PrP concentration by ELISA using previously published methods (Wenborn et al 2015). ICSM 35 and ICSM 18 antibodies were supplied by D-Gen Ltd, London. Numerous other anti-PrP antibodies from commercial or academic sources could be used for these purposes and there is no reliance on the particular properties of ICSM 35 and ICSM 18.
Validation	Validation is provided in Wenborn et al 2015 and references cited therein.

Eukaryotic cell lines

Policy information about cell lines

Cell line source(s)	PK1/2 cells (an established cell line; D-Gen Ltd London) were used to measure RML prion infectivity in cell culture using

	previously published methods (Wenborn et al 2015; Schmidt et al 2015).
Authentication	PK1/2 cells are a subclone of N2a cells that were obtained from, and authenticated by, the American Type Culture Collection (ATCC).
Mycoplasma contamination	PK1/2 cells have been tested negative for mycoplasma contamination.
Commonly misidentified lines (See ICLAC register)	None

Animals and other organisms

Policy information about studies involving animals; ARRIVE guidelines recommended for reporting animal research

Laboratory animals	200 female CD1 mice, ~200 days old, terminally-infected with RML prions.
Wild animals	Not applicable
Field-collected samples	Not applicable
Ethics oversight	Frozen brains from mice with clinical prion disease were used to generate purified prion samples. These brain samples were generated by us as part of a previous study (Wenborn et al 2015) in which work with animals was performed in accordance with licences approved and granted by the UK Home Office (Project Licences 70/6454 and 70/7274) and conformed to University College London institutional and ARRIVE guidelines. All experimental protocols were approved by the Local Research Ethics Committee of UCL Queen Square Institute of Neurology/National Hospital for Neurology and Neurosurgery.

Note that full information on the approval of the study protocol must also be provided in the manuscript.
